# A new Rogue-like *Escherichia* phage UDF157lw to control *Escherichia coli* O157:H7

**DOI:** 10.3389/fmicb.2023.1302032

**Published:** 2024-01-22

**Authors:** Yen-Te Liao, Kan-Ju Ho, Yujie Zhang, Alexandra Salvador, Vivian C. H. Wu

**Affiliations:** Produce Safety and Microbiology Research Unit, U.S. Department of Agriculture, Agricultural Research Service, Western Regional Research Center, Albany, CA, United States

**Keywords:** lytic bacteriophage, *Rogunavirus* genus, *E. coli* O157:H7, depolymerase enzyme, anti-biofilm activity

## Abstract

**Introduction:**

Shiga toxin-producing *Escherichia coli* (STEC) O157:H7 is one of the notorious foodborne pathogens causing high mortality through the consumption of contaminated food items. The food safety risk from STEC pathogens could escalate when a group of bacterial cells aggregates to form a biofilm. Bacterial biofilm can diminish the effects of various antimicrobial interventions and enhance the pathogenicity of the pathogens. Therefore, there is an urgent need to have effective control measurements. Bacteriophages can kill the target bacterial cells through lytic infection, and some enzymes produced during the infection have the capability to penetrate the biofilm for mitigation compared to traditional interventions. This study aimed to characterize a new *Escherichia* phage vB_EcoS-UDF157lw (or UDF157lw) and determine its antimicrobial efficacy against *E. coli* O157:H7.

**Methods:**

Phage characterization included biological approaches, including phage morphology, one-step growth curve, stability tests (pH and temperature), and genomic approaches (whole-genome sequencing). Later, antimicrobial activity tests, including productive infection against susceptible bacterial strains, *in vitro* antimicrobial activity, and anti-biofilm, were conducted.

**Results:**

UDF157lw is a new member of the phages belonging to the *Rogunavirus* genus, comprising a long and non-contractile tail, isolated from bovine feces and shares close genomic evolutionary similarities with *Escherichia* phages vB_EcoS-BECP10 and bV_EcoS_AKS96. When used against *E. coli* O157:H7 (ATCC35150), phage UDF157lw exhibited a latent period of 14 min and a burst size of 110 PFU per infected cell. The phage remained viable in a wide range of pH values (pH 4–11) and temperatures (4–60°C). No virulence genes, such as *stx*, lysogenic genes, and antibiotic resistance genes, were found. Phage UDF157lw demonstrated high infection efficiencies against different *E. coli* O157:H7 and generic *E. coli* strains. In addition, UDF157lw encoded a unique major tail protein (ORF_26) with prominent depolymerase enzyme activity against various *E. coli* O157:H7 strains, causing large plaque sizes. In contrast to the phage without encoding depolymerase gene, UDF157lw was able to reduce the 24-h and 48-h *E. coli* O157:H7 biofilm after 1-h phage treatment.

**Discussion:**

The findings of this study provide insights into a new member of the *Rogunavirus* phages and demonstrate its antimicrobial potential against *E. coli* O157:H7 *in vitro*.

## 1 Introduction

Shiga toxin-producing *Escherichia coli* (STEC) contains several well-known serogroups, including O157 and the top six non-O157, and has contributed to numerous foodborne outbreaks, causing 265,000 illnesses and 3,600 hospitalizations annually in the United States (CDC, 2006). These pathogens are commensal to cattle in the gastrointestinal tracts, and thus, some serogroups, particularly O157, have been linked to numerous cases of meat contamination since 1982, causing bloody diarrhea and hemolytic uremic syndrome among the infected people (Riley et al., 1983). In the current times, the contamination of the pathogenic bacteria occurs more frequently in leafy greens, with more than 18% of the produce-associated foodborne outbreaks caused by STEC in the United States (Tack et al., 2021), including a recent *E. coli* O157:H7 outbreak on spinach (https://www.cdc.gov/ecoli/2021/o157h7-11-21/index.html#print). The surge of these bacterial pathogens in produce likely derives from cross-contamination or direct contact with the contaminants, such as contaminated irrigation, soils, and animal feces, primarily from the pre-harvest environment (Lacombe et al., 2022). Therefore, several chemical sanitizers, such as sodium hypochlorite, sodium chlorite, and peroxyacetic acid, have been utilized to remove and control various foodborne pathogens, including *E. coli* O157:H7, in the food industry; however, outbreaks with leafy green products related to *E. coli* O157:H7 reoccurred even after these intervention measures were put in place (Lacombe et al., 2022).

Many foodborne pathogens, including *E. coli* O157:H7, can form biofilms, a group of bacterial cells embedded in a matrix of extracellular polymeric substances (EPS) with increasing adhesion to different types of surfaces (Galié et al., 2018). As a result, biofilms are not only difficult to remove but can also enhance the antimicrobial tolerance of bacterial pathogens, particularly to chemical sanitizers, resulting in common but critical food safety issues in most processing plants (Chitlapilly et al., 2020). Since *E. coli* O157:H7 has a low infection dose (Rahal et al., 2012) and can persist on a variety of surfaces (Marouani-Gadri et al., 2010; Kuruwita et al., 2022), biofilm formation of the pathogens poses an increasing threat in jeopardizing the microbiological safety of foods. In the food industry, physical interventional methods, such as hot water and ultrasonication, are usually used in addition to chemical sanitizers, the same as those for general sanitation, such as sodium hypochlorite, sodium chlorite, and peroxyacetic acid, to control biofilm formation (Galié et al., 2018). Even so, the unique structural characteristic of biofilm improves the possibility of resistance to these antimicrobial methods. Thus, there is an urgent need to develop new antimicrobial measurements to improve the existing sanitation system to resolve the biofilm problem, particularly with *E. coli* O157:H7.

Bacteriophages (or phages) are viruses of bacteria and the natural predators ubiquitous in the biosphere (O'Sullivan et al., 2019). Upon contact with their bacterial hosts, phages bind to specific receptor proteins on the bacterial cell membranes to initiate infection, thereby contributing to host-specific traits (Nobrega et al., 2018). Due to the nature of the lytic cycle, the phages utilize bacterial machinery to produce viral particles and lyse the bacterial cells to release the phage progenies (Clokie et al., 2011). With the increasing antibiotic resistance issues, (lytic) phage application is gradually re-recognized as an alternative antimicrobial intervention to combat antibiotic-resistant strains (Kahn et al., 2019; O'Sullivan et al., 2019). Although phage treatment contributes to bacterial resistance to the phage infection, the phage-resistant strains do not usually present a greater risk and could be more susceptible to the same antimicrobial interventions (León and Bastías, 2015; Pinto et al., 2021). Most of all, Generally Recognized As Safe (GRAS) has been granted for various phage products by the United States Food and Drug Administration (FDA); the regulation ensures the safety of the phage-based interventional technology, such as direct application on food or food-contact surface, thus making it more acceptable in the food industry (Pinto et al., 2020; Vikram et al., 2020).

Lytic phages were found to have mitigating effects against biofilm formation from the study of T4 phages ~30 years ago (Doolittle et al., 1995). Unlike chemical sanitizers, phages can penetrate biofilm for control, and some sequential enzymes produced during phage infection also harbor anti-biofilm activities to improve biofilm eradication (Łusiak-Szelachowska et al., 2020). Although many phages have been characterized and examined for biofilm control, those specific to *E. coli* O157:H7 with anti-biofilm potential are relatively scarce (Partanen et al., 2010; Połaska and Sokołowska, 2019). Therefore, this study aimed to characterize a new phage, *Escherichia* phage vB_EcoS-UDF157lw (or UDF157lw), via biological and genomic approaches and determine its antimicrobial potential against *E. coli* O157:H7 biofilm.

## 2 Materials and methods

### 2.1 Isolation of phages

*Escherichia* phage vB_EcoS-UDF157lw (or UDF157lw) was isolated from a bovine fecal sample using *E. coli* O157:H7 as a bacterial host and subsequently subjected to purification through the methods previously described by Liao et al. (2020). Phage was propagated by mixing 50 μl of phage lysate (~10^6^ PFU/ml) with 100 μl of an overnight culture of *E. coli* O157:H7 (ATCC35150) per plate using a double-layer plaque assay. After overnight incubation at 37°C, the plates with bacterial lysis were added with 10 ml of sodium chloride and magnesium sulfate (SM) buffer (Teknova Inc., Hollister, CA, United States) and then incubated at room temperature for 6 h with shaking to elute phage UDF157lw. Later, the eluted phage solution was centrifuged at 8,000 × *g* for 5 min, followed by filtration through a 0.22-μm filter membrane to remove bacteria prior to downstream analysis.

### 2.2 Bacterial strains

A panel of bacterial strains consisting of the top six non-O157 STEC (O26, O45, O103, O111, O121, and O145), *E. coli* O157:H7, generic *E. coli*, and *Salmonella enterica* strains (Supplementary Table S1) acquired from the Produce Safety and Microbiology (PSM) Research Unit at the United States Department of Agriculture (USDA), Agricultural Research Service (ARS), Western Regional Research Center (WRRC), Albany, CA, United States, were used for the host range and antimicrobial tests in this study. *Escherichia coli* O157:H7 (ATCC35150) was used for phage propagation, quantification, and the one-step growth curve. Fresh bacterial cultures were prepared by inoculating one 10-ml tryptic soy broth (TSB; Becton Dickinson, Sparks, MD) with 1 μl loopful of individual strains and incubating overnight at 37°C before use.

### 2.3 Genomic analysis

Phage UDF157lw was purified through a cesium chloride (CsCl) gradient centrifugation to remove bacterial debris as previously described by Liao et al. (2022b). Phage DNA was extracted using a Norgen phage DNA extraction kit (Thorold, ON, Canada), followed by DNA library preparation before sequencing, as previously described by Zhang et al. (2021). Later, the phage DNA libraries were sequenced using a MiSeq Reagent Kit v2 (500-cycle) on the MiSeq platform (Illumina, San Diego, CA, United States), generating ~5 million 2 × 250-bp paired-end reads. Raw sequence reads were subjected to quality checks using FASTQC v.0.12.1 and trimming poor quality reads via Trimmomatic v.0.38 with the setting of Q30. *De novo* assembly was conducted on the quality reads using Unicycler v.0.5.0 with the default settings. The resulting contig with an N50 contig length of 46,392 bp (also the largest contig) was confirmed as a phage sequence via BLASTn search. Later, the packaging mechanisms and termini of the phage genome were predicted using PhageTerm Galaxy v.1.0.12 (Garneau et al., 2017). The re-organized phage sequence from the PhageTerm analysis was annotated using the Prokka pipeline Galaxy 1.13 (Seemann, 2014) with the default settings. Subsequently, the annotation was confirmed with blastp against the Universal Protein Resource (UniProt) database (Bairoch et al., 2005) and manually curated using Geneious Prime (v2023.2 Biomatters, New Zealand). The predicted tRNAs in the phage genome were confirmed using the tRNAscan-SE (v2.0) server (Lowe and Chan, 2016). The final phage sequence of UDF157lw, obtained from the PhageTerm analysis, was subjected to the screening of virulence and antibiotic resistance genes in phage genomes using Webservers VirulenceFinder v2.0 (https://cge.food.dtu.dk/services/VirulenceFinder/; accessed on 07 September 2023) (Malberg Tetzschner et al., 2020) and ResFinder v4.1 (https://cge.food.dtu.dk/services/ResFinder/; accessed on 07 September 2023) (Bortolaia et al., 2020), respectively, with the default settings. The final annotated sequence of UDF157lw was deposited in the National Center for Biotechnology Information (NCBI) database, with accession number OQ243221. A genome map of UDF157lw was performed using the CGview server beta (https://proksee.ca/; accessed on 29 August 2023).

### 2.4 Comparative genomics

The evolutionary tree based on the whole-genome sequence of UDF157lw with its close-related reference phages was analyzed using the Virus Classification and Tree Building Online Resource (VICTOR) webserver at the nucleic acid (D0) and amino acid (D6) levels (Meier-Kolthoff and Göker, 2017). The conservative (core) genes of UDF157lw were analyzed with the close-related reference phages using CoreGenes3.5 (Mahadevan et al., 2009). Phylogenetic analyses of the amino acid sequences were conducted on the major tail protein (ORF_26), tail fiber protein (ORF_35), holin (ORF_60), and lysozyme (ORF_61) using the methods previously described by Liao et al. (2022b). In brief, the amino acid sequences were aligned using ClustalW (version 1.2.3) from Geneious Prime. The phylogenetic trees were performed using the MEGA11 program with the maximum likelihood method and 500 bootstrap replications (Jones et al., 1992).

### 2.5 Transmission electron microscopy

CsCl-purified phage UDF157lw was subjected to negative staining and examined in a transmitted electron microscope (TEM, FEI Tecnai G2), as previously described by Liao et al. (2022b). In brief, 5 μl of the CsCl-treated phage was placed on a discharged 300 mesh carbon/formvar grid for 2-min absorption, followed by washing with distilled water and removing extra liquid. Subsequently, 5 μl of 1% uranyl acetate stain was added to the grid for negative staining. After drying, the sample was ready to be viewed under transmission electron microscopy (TEM).

### 2.6 One-step growth curve

The one-step growth curve of phage UDF157lw was determined using *E. coli* O157:H7 (ATCC35150) based on the previous method with subtle modifications (Liao et al., 2022b). In brief, 0.2 ml of fresh overnight culture in TSB was sub-cultured in 19.8 ml of sterile TSB and incubated for 2 h at 37°C to reach the log phase of bacterial growth. Later, UDF157lw was added to the log-phase bacterial solution (MOI of 0.01) with CaCl_2_ at 10 mM and incubated at 37°C for 10 min, allowing phage adsorption onto the bacterial membranes. The phage-bacterial mixture was centrifuged at 10,000 × *g* for 5 min at 4°C to discard the supernatant. After washing, the bacterial pellet was resuspended with 20 ml of fresh TSB before further 10-fold dilution (0.3 ml of resuspension in 29.7 ml of TSB). The sample was then incubated at 37°C for 40 min. Meanwhile, the phage-infected bacterial cells were measured prior to incubation (time 0) by combining 50 μl of the 30-ml phage-bacterial mixture (no filtration) with 100 μl of a newly cultured bacterium overnight to perform a double-layer plaque assay (Liao et al., 2022a). Subsequently, 1 ml of the phage-bacterial mixture was obtained every 5 min and filtered using a 0.22-μm filter membrane. The titers of phage UDF157lw at each time point were determined using the double-layer plaque assay. The experiment was conducted in three repeats to estimate the latent period—the time required to lyse the bacterial cell walls and release phage progenies—and the burst size of phage UDF157lw.

### 2.7 Stability tests (pH and temperature)

A range of pH values from pH 3 to 12 was used to test the stability of UDF157lw at 30°C for 20 h using the method as previously described with minor changes (Liao et al., 2022a). In brief, 100 μl of phage UDF157lw was added to 4.9 ml of SM buffer with the final pH values of 3, 4, 5, 7, 10, 11, and 12. Viable phage particles were determined against *E. coli* O157:H7 (ATCC35150) using the double-layer plaque assay.

For the temperature stability test, bulk phage lysate of UDF157lw was prepared by combining the original phage lysate with SM buffer at a 1:9 (v/v) ratio. Later, an aliquot of 1 ml of UDF157lw solution at pH 7 was dispensed in several sterile microcentrifuge tubes and subjected to refrigeration temperature (4°C) and heat treatments, including 30°C, 50°C, 60°C, 65°C, and 70°C, for 24 h. The temperatures covered were inclusive of the general conditions encountered in food-related environments. Phage titers were determined using the double-layer plaque assay.

### 2.8 Host range, productive infection, and plaque morphology

The host range test of UDF157lw against non-pathogenic *E. coli, E. coli* O157:H7, top six non-O157 STEC, and three *Salmonella* strains was determined using a spot test assay, as previously described by Liao et al. (2022b). The susceptible bacterial strains, with lysis, were then used to determine the productive infection of phage UDF157lw based on the efficiency of plating (EOP) assay (Liao et al., 2019). Briefly, fresh bacterial cultures were prepared in TSB at 37°C overnight and used for the quantification of UDF157lw by the double-layer plaque assay with diluted phage lysate using four successive dilutions (10^−3^ to 10^−7^). The plates were incubated at 37°C for 18 h. The experiment was conducted in three repeats. Generally, a high phage-producing efficiency had an EOP of 0.5 or more; a medium-producing efficiency had an EOP above 0.1 but below 0.5; a low-producing efficiency had an EOP between 0.001 and 0.1; and inefficient phage production was any value lower than 0.001. Later, a picture of each plaque assay plate per bacterial strain was taken using a digital camera for plaque morphology observation.

### 2.9 *In vitro* antimicrobial activity test

*Escherichia coli* O157:H7 (RM9995) was used to evaluate the antimicrobial activity of UDF157lw using the method previously described with subtle modifications (Liao et al., 2022a). In brief, the bacterial culture was prepared in 10 ml of TSB at 37°C for 18 h, followed by dilution in lysogeny broth (LB; Invitrogen, Carlsbad, CA, United States) to reach the final concentration of ~5 log CFU/ml for the experiment. Phage UDF157lw in SM buffer was added to a 4-ml bacterial solution at approximately MOIs of 10, 100, and 1,000. For the control, SM buffer, with the same volume as the phage used in the treatment, was also added to a 4-ml bacterial solution. The control and treatment groups were both incubated at 25°C, and the bacterial counts were quantified at various time points (0, 1, 2, and 4 h) during the incubation. Bacterial counts were quantified on Sorbitol MacConkey agar (BD, Franklin Lakes, NJ, United States) overlayered with thin Tryptic soy agar (TSA) (Thin Agar Layer Method, TAL) (Wu, 2008).

### 2.10 Antimicrobial activity against *E. coli* O157:H7 biofilm

Biofilm was formed and evaluated using the previous methods (Guo et al., 2017), with some modifications. *Escherichia coli* O157:H7 (RM9995) was prepared in 10 ml of TSB and incubated at 37°C for 20 h with shaking. The next day, the overnight culture was diluted 100 times in TSB and then dispensed with 1 ml of the bacterial solution in triplicate wells containing a sterile coverslip per well in a 24-well plate. An aliquot of 1 ml of fresh TSB was used as a negative control. The 24-well plates were incubated at 37°C for 24 h and 48 h without shaking. After incubation, free bacterial cells were removed via pipetting out the culture, followed by washing steps with 0.5 m of 0.1% peptone water twice to evaluate the biofilm formation with the following process using methanol and crystal violet. Meanwhile, phages—UDF157lw and phage A (a small plaque-producing phage)—were diluted in TSB to reach the target concentration (~8 log PFU/ml); an aliquot of 1 ml of individual phage solution was added to the wells containing biofilm in triplicates for 1-h incubation at 37°C without shaking. An aliquot of 1 ml of fresh TSB was added as the control group. After the treatment, the TSB, with or without phages, was discarded, followed by the washing process, as described earlier. Later, an aliquot of 0.5 ml of methanol was added to all wells and incubated at room temperature for 30 min. After methanol removal, plates were dried in the hood before adding 0.05% crystal violet to stain the bacterial cells in the wells for 30-min incubation at room temperature. Subsequently, the wells were washed gently with 0.5 ml of 0.1% peptone water twice following crystal violet removal. After drying the plates at room temperature, 0.5 ml of 33% (v/v) glacial acetic acid was added to the wells to release the binding crystal violet from the strained biofilm. The optical density (OD) at a wavelength of 590 nm was measured using a spectrophotometer (BioTek, Winooski, WT, United States). The biofilm was determined as a strong (4 × ODnc ≤ OD), moderate (2 × ODnc < OD ≤ 4 × ODnc), or weak (ODnc < OD ≤ 2 × ODnc) biofilm formation or non-biofilm formation (OD ≤ ODnc) according to a previous description (Stepanovic et al., 2000). ODnc was the OD measurement from the negative control containing only TSB. Phage A, without encoding the depolymerase enzyme, produced small plaques (Supplementary Figure S2) and was used to compare the anti-biofilm activity with phage UDF157lw.

### 2.11 Statistical analysis

Experiments subjected to statistical analysis were conducted in three individual repeats. The quantification of phages was calculated as PFU/ml, with a logarithmical conversion for statistical analysis. The stress effect of pH (pH 4–10) and temperature test on UDF157lw and *in vitro* antimicrobial activities of different MOIs at each time point were determined by the one-way analysis of variance (ANOVA), with a statistical significance at a 5% level.

## 3 Results

### 3.1 Genomic characteristics of UDF157lw

**Figure 1:**
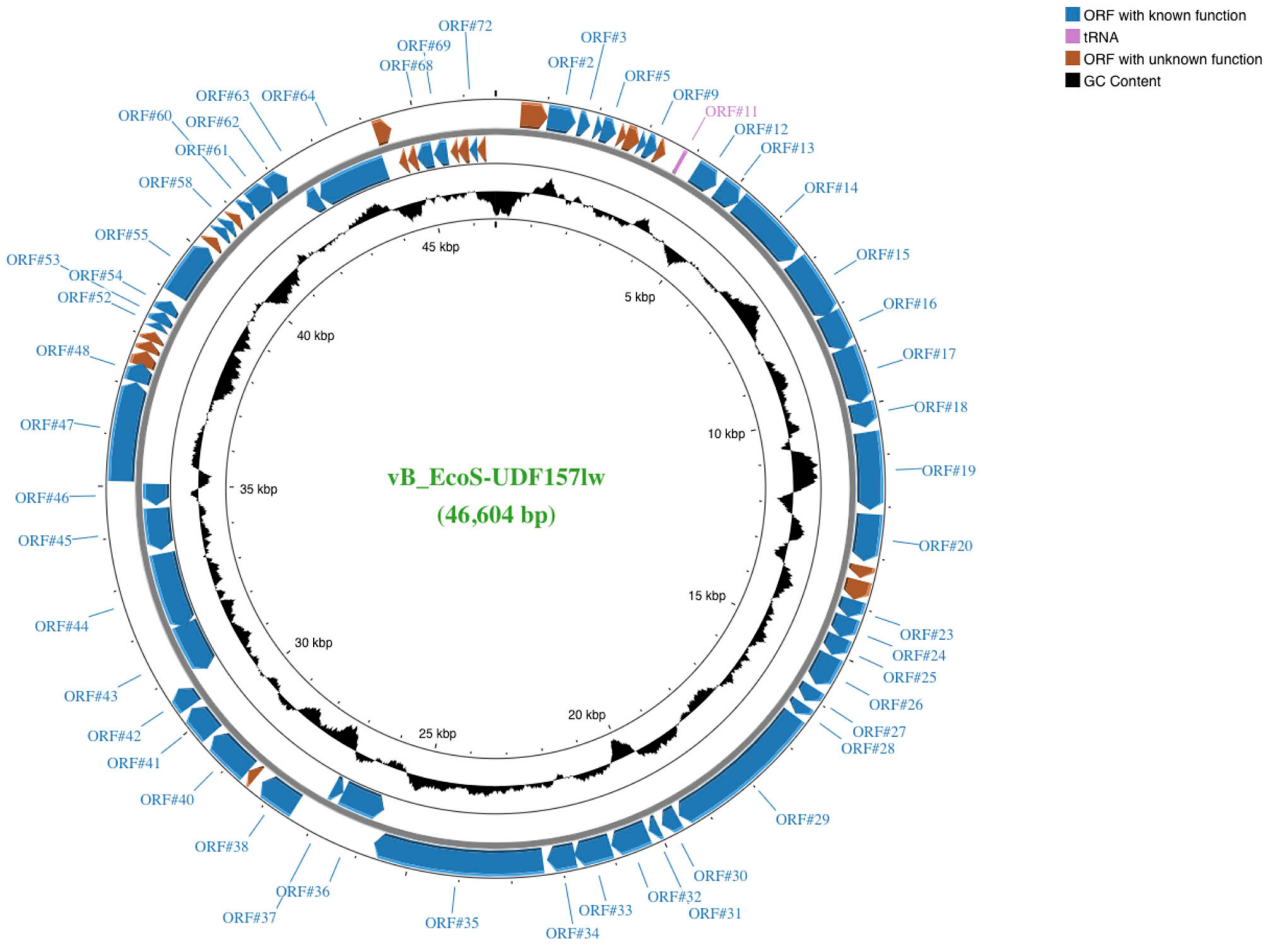
A genome map of *Escherichia* phage vB_EcoS-UDF157lw (or UDF157lw) generated using the CGview server beta. ORFs with known and unknown predicted functions are colored, and the detailed annotated information can be found in Supplementary Table S2. The center of the genome map provides % GC content (black).

UDF157lw had double-stranded DNA and a relatively small genome, with a size of 46,604 bp and a GC content of 44% (Figure 1). The phage contained 73 open reading frames (ORFs), including 54 annotated with known functions and 1 tRNA (Arg; Supplementary Table S2). The ORFs with known predicted functions were associated with structural proteins—such as capsid and tail structures—bacterial host recognition (tail fibers), host lysis (holin, lysozyme, and spanin), phage DNA replication, and host cell regulation and metabolism (Supplementary Table S2). Most importantly, UDF157lw did not contain virulent genes, such as *stx*, lysogenic genes, and antibiotic resistance genes, in the genome. In addition, phage UDF157lw was predicted to have a headful DNA packaging mechanism with a preferred packaging (pac) site (Oliveira et al., 2013).

**Figure 2:**
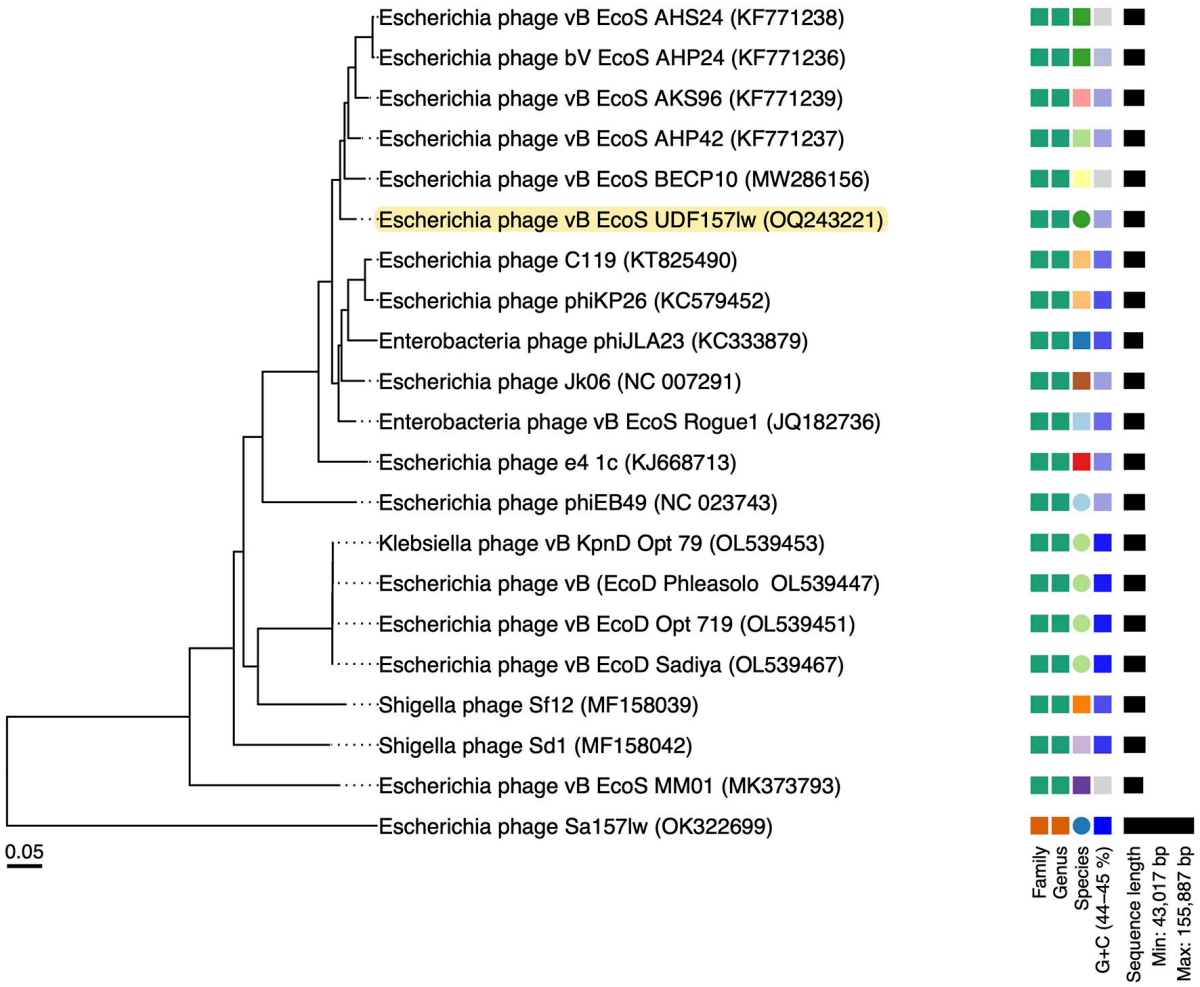
A whole-genome sequence-based phylogenetic tree constructed using VICTOR (formula d0) on UDF157lw, other *Caudoviricetes* phage Sa157lw, and the close-related reference phages belonging to the *Rogunaviru* genus under the *Drexlerviridae* family. The family, genus, and species are classified into different clusters with colors based on the VICTOR analysis. Genomic GC content and sequence length are represented in the hue of color and horizontal black lines, respectively, on the right side of the tree.

The results of the VICTOR analysis showed that phage UDF157lw was at its own leaf at the nucleic acid level but classified at the same genus level as other reference phages belonging to the *Rogunavirus* genus (Figure 2). However, based on the amino acid sequence analysis, UDF157lw shared a close relationship with *Escherichia* phage vB_EcoS-BECP10 (Supplementary Figure S1). In comparison to two *Rogunavirus* reference phages with the highest BLASTn similarity, UDF157lw shared an average nucleotide identity (ANI) of 97.15% (89.2% query coverage) and 95.98% (89.67% query coverage) with *Escherichia* phage vB_EcoS-BECP10—initially classified in the *Jk06likevirus* genus (Park and Park, 2021)—and *Escherichia* phage bV_EcoS_AKS96, respectively, calculated by JSpeciesWS (Richter et al., 2016).

**Figure 3:**
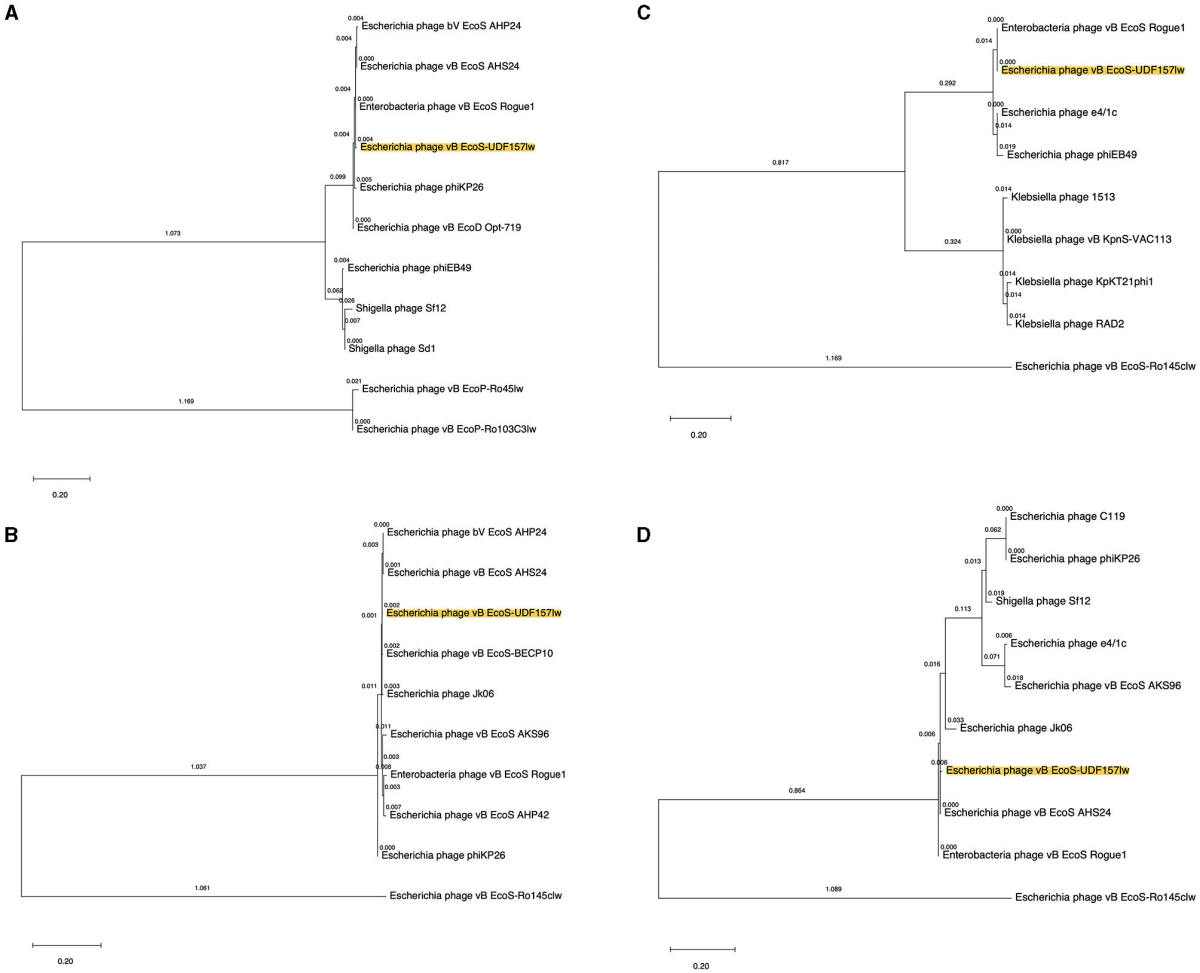
Phylogenetic tree of the amino acid sequences of major tail protein (ORF_26) **(A)**, tail fiber protein (ORF_35) **(B)**, holin (ORF_60) **(C)**, and lysozyme (ORF_61) **(D)** from UDF157lw (with yellow highlight), other *Caudoviricetes* phages, and the reference phages belonging to the *Rogunaviru* genus. The tree is drawn to scale, with branch lengths measured in the number of substitutions per site (above the branches).

Phylogenetic analyses were conducted at the amino acid level on the ORFs encoding the proteins related to bacterial host recognition and bacterial cell lysis enzymes. UDF157lw contained a unique major tail protein with potential depolymerase activity, encoded by ORF_26, compared to the reference phages (Figure 3A). ORF_35 coded for a tail fiber protein in UDF157lw shared a close evolutionary relationship with the counterfeit in *Escherichia* phage vB_EcoS-BECP10 (Figure 3B). For holin and lysozyme, UDF157lw shared a high amino acid identity with that of *Escherichia* phage vB_EcoS_Rogue1 and *Escherichia* phage vB_EcoS_AHS24, respectively (Figures 3C, D).

### 3.2 Phage morphology

**Figure 4:**
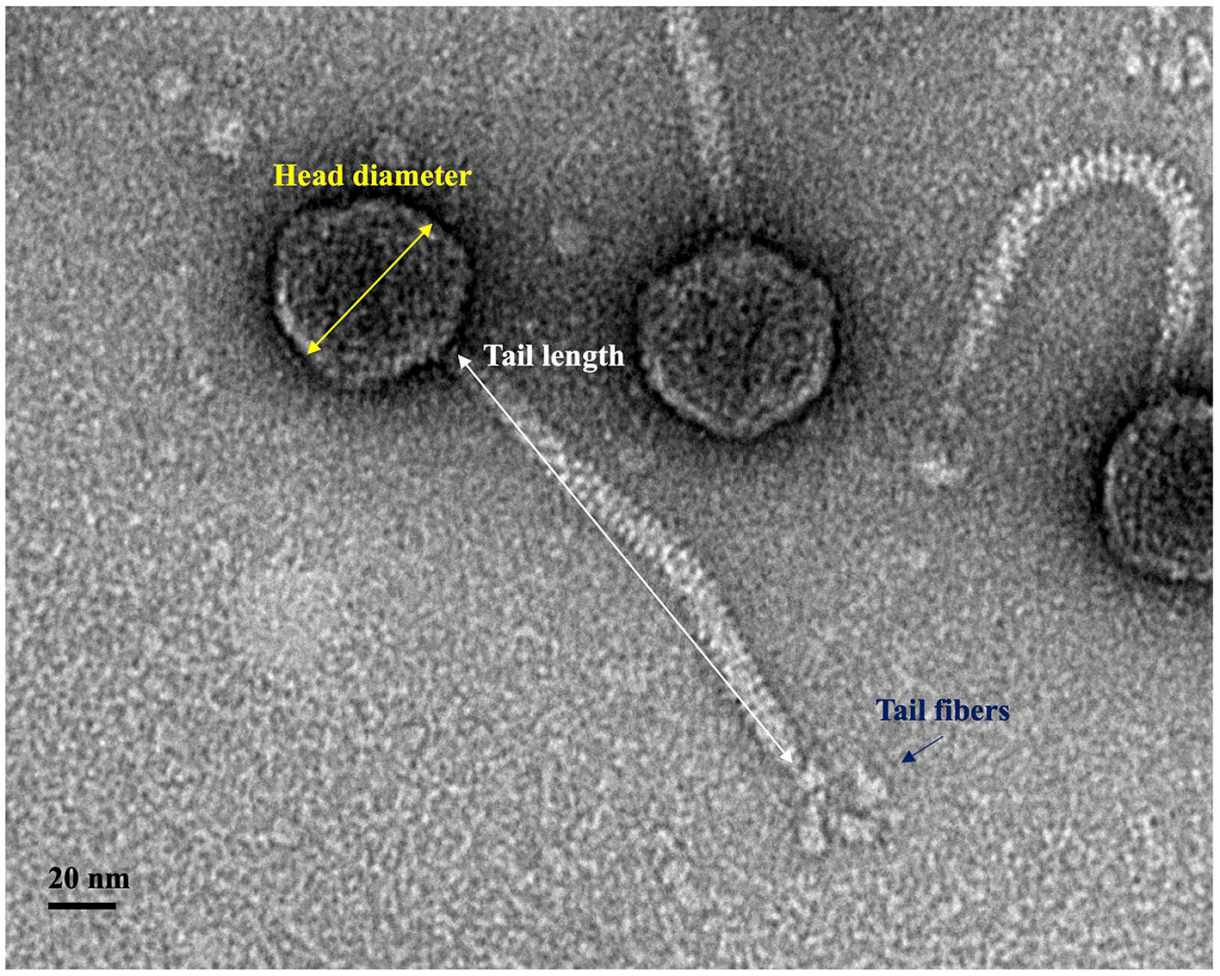
Transmission electron microscopy image of phage UDF157lw with an icosahedral capsid (55 ± 3 nm in diameter), a non-contractile tail (177 ± 3 nm in length), and tail fibers.

UDF157lw had an icosahedral head with ~55 ± 3 nm in diameter and a long non-contractile tail of 177 ± 3 nm in length, demonstrating the *Siphoviridae* morphology (Figure 4). The phage also had visible star-like tail fibers at the end of the tail structure, and the morphology was similar to that of T1-like phages (Kropinski et al., 2012).

### 3.3 One-step growth curve and stability tests

**Figure 5:**
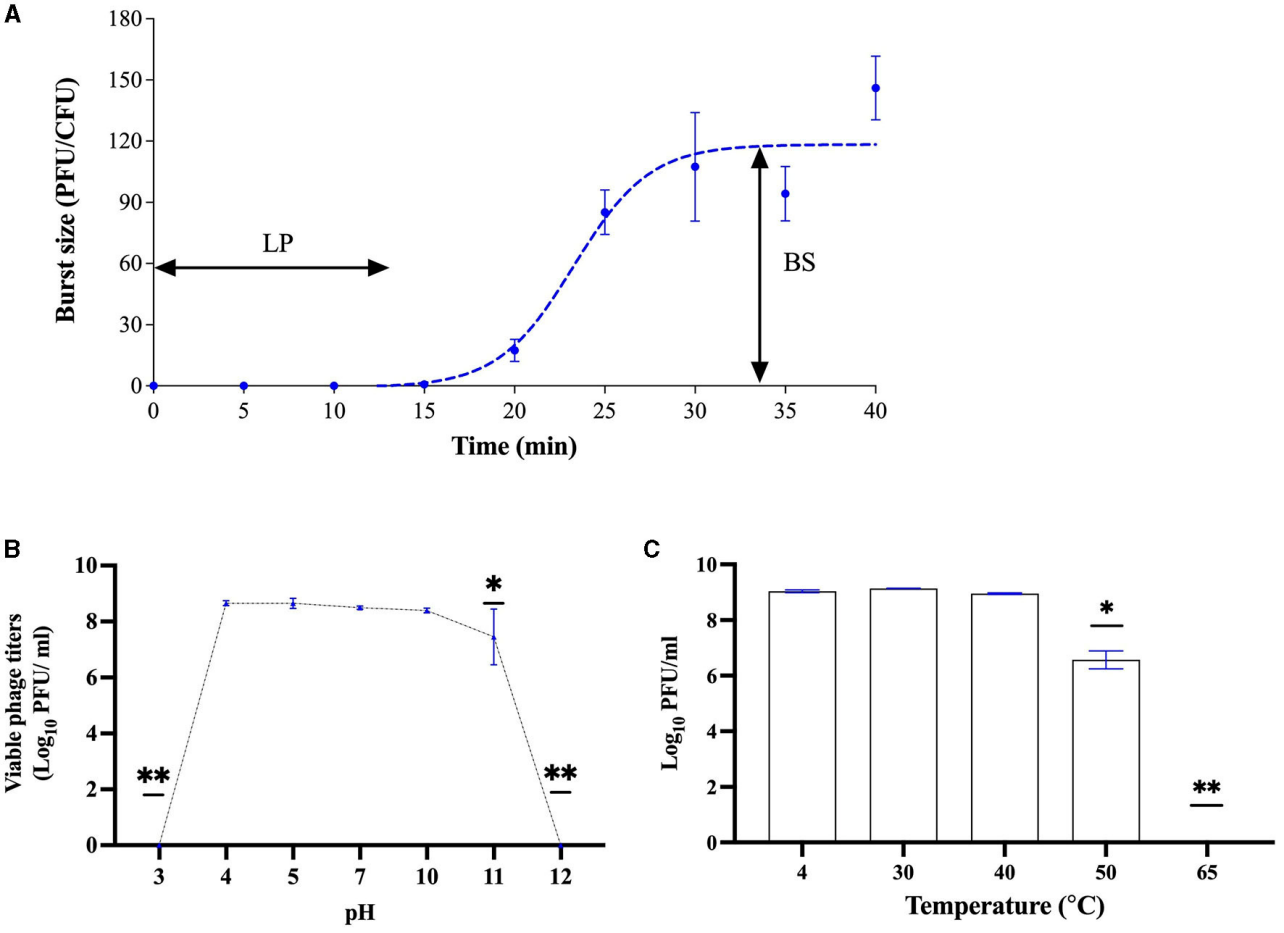
Biological characteristics of phage UDF157lw, including a one-step growth curve on *Escherichia coli* O157:H7 ATCC35150 with a latent period (LP) of 14 min and an average burst size (BS) of 110 PFU per infected cell **(A)**, various pH stability at 30°C for 20 h **(B)**, and temperature stability for 24 h **(C)**. For stability tests, the means of phage titers that contain different numbers of asterisks differ (*P* < *0.05*). The error bars show the standard error of the mean (SEM).

For the growth factor, UDF157lw had a latent period of 14 min against *E. coli* O157:H7 (ATCC35150) with a burst size of 110 PFU per infected cell (Figure 5A). With regard to stress stability, the phage could sustain a wide range of pH values from pH 4–11, pertaining to a similar level of pH values from pH 4–10 but slightly dropping at pH 11. However, UDF157lw was at the non-detected level at pH 3 or 12 (Figure 5B). For the temperature test, phage UDF157lw was stable from 4 to 50°C but significantly decreased in titer at 60°C by ~2 log and further dropped to a non-detectable level at 65°C (Figure 5C).

### 3.4 Host range, productive infection, and plaque morphology

**Figure 6:**
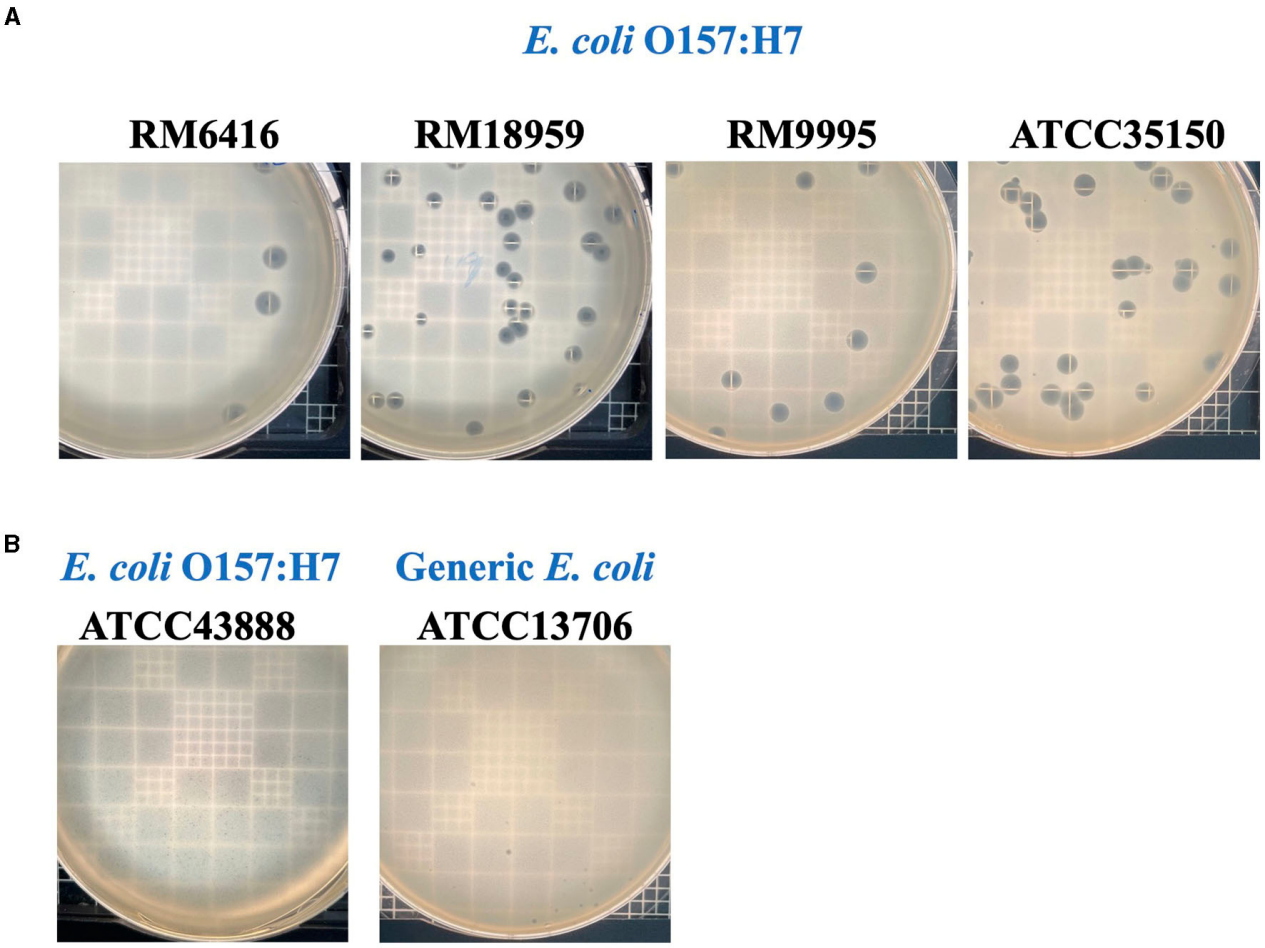
Plaque morphologies of phage UDF157lw producing large plaque sizes **(A)** against different *Escherichia coli* O157:H7 and small plaque sizes **(B)** against *E. coli* O157:H7 (ATCC43888) and generic *E. coli* (ATCC13706) strains on plaque-assay plates with top soft agar.

Phage UDF157lw had a narrow host range and showed lytic infection targeting various *E. coli* O157:H7 strains and generic *E. coli* ATCC13706 (Table 1). Based on the EOP results, UDF157lw showed low production infection on a generic *E. coli* compared to the primary *E. coli* O157:H7 strain (ATCC35150). However, the phage demonstrated medium to high production infection against most *E. coli* O157:H7 strains, with the EOP ranging from 0.25 to 1.1, except for the *E. coli* O157:H7 ATCC43888 (inefficiency infection). Interestingly, among all the plaque assay plates, UDF157lw produced a halo on each plaque, causing large plaques against most *E. coli* O157:H7 strains (Figure 6A), except for *stx*-deficient *E*. coli O157:H7 (ATCC43888) and generic *E. coli* (Figure 6B).

**Table 1:** Host range and efficiency of plating (EOP) of phage UDF157lw against different *Salmonella enterica* serovars and Shiga toxin-producing *Escherichia coli* serogroups.

**Strains**	**Strain Ref. No**.	EOP^*^
Non-O157 STEC	STEC O26, O45, O103, O111, O121, and O145	R
STEC O157	*E. coli* O157:H7 (RM18959)	1.1
	*E. coli* O157:H7 (ATCC35150)	H
	*E. coli* O157:H7 (ATCC43888)	< 0.001
	*E. coli* O157:H7 (RM9995)	1.63
	*E. coli* O157:H7 (RM6416)	0.32
	*E. coli* O157:NM (RM11781)	0.25
Generic *E. coli*	ATCC13706	0.06
	ATCC15597	R
	TVS 353	R
*Salmonella*	*Salmonella* Typhimurium 14028	R
	*Salmonella* Infantis BAA-1675	R

^*^EOP was calculated by the ratio of phage titer on a test bacterium versus the primary bacterial host. High production efficiency is EOP ≥0.5, medium production efficiency is 0.5 > EOP ≥ 0.1, low production efficiency is 0.1 > EOP > 0.001, and the inefficiency of phage production is EOP ≤ 0.001.

H means the host strain used for the phage isolation.

R means the bacterial strain is resistant to the phage infection.

### 3.5 *In vitro* antimicrobial and anti-biofilm activities of UDF157lw

**Figure 7:**
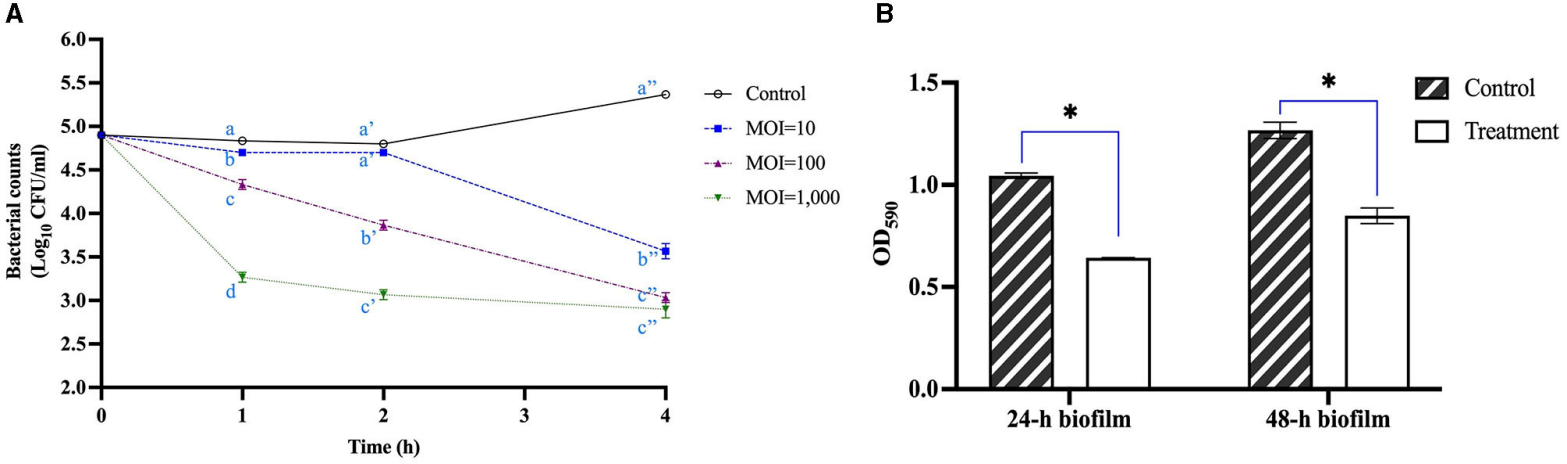
Antimicrobial activity of phage UDF157lw with MOI of 10, 100, and 1,000 against *Escherichia coli* O157:H7 (RM9995) in lysogeny broth at 25°C for 4 h **(A)**, and 24-h and 48-h *E. coli* O157:H7 (RM9995) biofilm at 10^8^ PFU/ml at 37°C for 1 h based on the bacterial optical density at 590 nm (OD_590_) **(B)**. The control group only contains bacterial cultures. For antimicrobial activity tests, means of phage titers within each time point lacking common letters (a, b, and c; a′, b′, c′, and d′; or a″, b″, and c″) differ (*P* < *0.05*). The error bars show the SEM. Asterisk indicates a significant difference at *P* < 0.05 between the control and treatment groups.

The antimicrobial activity of UDF157lw against *E. coli* O157:H7 (RM9995) was evaluated in the LB broth. The results showed that the bacterial levels were significantly reduced after 1 h of the phage treatment at an MOI of 10 compared to the control group and remained unchanged for 1 h until further reduction at the 4-h time point by 1.8 log (Figure 7A). Additionally, the treatment group of MOI = 100 constantly mitigated the bacterial population and reduced bacterial levels by 2.3 log after 4-h treatment. Moreover, the bacteria treated with phage UDF157lw at a MOI of 1,000 had the best antimicrobial effect among all MOIs at 1 h in reducing the cells by 1.4 log (compared to the control), even though the lytic activity against *E. coli* O157:H7 slowed down after the 1-h treatment.

Regarding the potential anti-biofilm activity test, the results showed that the OD_590_ values of 24- and 48-h *E. coli* O157:H7 biofilms, treated with UDF157lw at ~8 log PFU/ml for 1 h, were significantly reduced compared to the control groups (Figure 7B). Conversely, the OD_590_ value of the same 24-h biofilm treated with another *E. coli* O157:H7-infecting phage A, which did not show depolymerase enzyme activity or encode the related genes (data not shown), thus causing small plaques, remained at a similar level as the control group (*P* > *0.05*) (Supplementary Figure S2). These results demonstrated that phage UDF157lw with depolymerase enzyme activity could remove *E. coli* O157:H7 cells from a 24- or 48-h biofilm in a 24-well plate system.

## 4 Discussion

Phage application is a promising and alternative antimicrobial invention solution for bacterial resistance to antibiotics and chemical antimicrobials. Although lytic phages have demonstrated their antimicrobial activity against biofilm (Hyla et al., 2022), the mitigating effects vary from phage to phage. The current study focused on characterizing a new *Rogunavirus* phage for its antimicrobial effects on *E. coli* O157:H7. Phage UDF157lw harbors genomic features, including ~45 kb genome size, ~44% GC content, and 1 tRNA, and a morphology similar to several reference phages belonging to the *Rogunavirus* genus (Kropinski et al., 2012; Niu et al., 2014). Furthermore, according to the International Committee on Taxonomy of Virus (ICTV) taxonomy guideline (Turner et al., 2021), the comparative genomics of this study suggest that phage UDF157lw is a new member belonging to the *Rogunavirus* genus under the *Drexlerviridae* family. The phages classified in the *Rogunavirus* genus, previously known as the *Jk06likevirus* genus, render lytic infection specific to *E. coli* O157:H7; however, the receptor-binding proteins of this group of phages are not well discovered (Niu et al., 2014). In addition, the narrow host range of these phages containing a long and non-contractile tail, known as *Siphoviridae* morphology, is primarily affected by the tail structures, such as tail fiber or tail spike proteins, compared to *Myoviridae* phages, which encode a long and contractile tail with a broad host range (Chibani-Chennoufi et al., 2004; Sørensen et al., 2021). Similarly, UDF157lw has a narrow host spectrum, specifically lytic against *E. coli* O157:H7 strains of various sources and generic *E. coli* ATCC13706. Although showing overall high amino acid similarity with phage vB_EcoS-BECP10, UDF157lw had a slightly different host recognition activity from vB_EcoS-BECP10, which targeted only *E. coli* O157:H7 (Park and Park, 2021). The divergence is likely because UDF157lw—without encoding tail spike protein—might be using tail fiber proteins to target other outer membrane proteins or different parts of lipopolysaccharides other than just O-antigen residue on the bacterial host as phage vB_EcoS-BECP10 does with a tail spike protein (Park and Park, 2021). Interestingly, among all *E. coli* O157:H7 strains, UDF157lw only displayed inefficient infection against *stx*-deficient *E. coli* O157:H7 (ATCC43888). The phenomenon could be due to the fitness properties of ATCC43888 being different from most *E. coli* O157:H7 harboring *stx* genes (Uhlich et al., 2019), possibly altering phage receptor-binding proteins (Knecht et al., 2020) or increasing host defense mechanisms interfering with phage DNA replication during phage infection (Howard-Varona et al., 2018).

In terms of growth factors, phage UDF157lw has a shorter latent period (14 min) than a polyvalent phage Sa157lw (30 min), which can infect *E. coli* O157:H7 and *Salmonella* strains (Liao et al., 2022b). The holin protein is one of the factors mediating the latent period (Abedon et al., 2001) and is encoded in the UDF157lw genome, potentially contributing to a rapid phage-releasing time compared to Sa157lw without the protein (Liao et al., 2022b). UDF157lw has a large burst size of 110 PFU per infected cell, which is one of the advantages of being used as a biocontrol agent because a high level of phage progenies will be produced per infection cycle for the subsequent infection (Nilsson, 2014). Like most *E. coli* phages (Merabishvili et al., 2013; Son et al., 2018; Liao et al., 2022b), UDF157lw can maintain infectivity in a wide range of pH values (4–10) and temperatures of up to 60°C. These biological features ensure the effectiveness of phage UDF157lw when applied in an environment exposed to various conditions.

UDF157lw can produce a large plaque consisting of a clear center surrounded by a halo zone in a top soft agar overlay. The halo zone is developed by an enzyme activity known as the depolymerase enzyme, which is initially found in *Podoviridae* phages targeting receptor-binding proteins of their bacterial hosts through the phage tail spikes and has anti-biofilm activity (Knecht et al., 2020). Meanwhile, similar enzymatic action has been observed in other phage morphology types—*Siphoviridae* and *Myoviridae—*targeting different parts of bacterial membrane structures, such as capsular polysaccharides and exopolysaccharides (Knecht et al., 2020). UDF157lw has a unique major tail protein, also expressing depolymerase enzyme activity in addition to its structural function, compared to that from the close-related reference phages; moreover, the enzyme activity is selectively active against *E. coli* O157:H7 strains except for *stx*-deficient *E. coli* O157:H7 (ATCC43888) and generic *E. coli* ATCC13706. The findings suggest that the expression of depolymerase enzyme activity in UDF157lw is not solely related to the binding of O-antigen located at lipopolysaccharides on *E. coli* O157:H7 membranes (Knecht et al., 2020). Further study is needed to evaluate the association between the receptor proteins on the susceptible bacterial membranes and the depolymerase enzyme activity.

The antimicrobial activity of UDF157lw with an MOI of 10 did not reduce *E. coli* O157:H7 cells between 1 h and 2 h in LB broth at 25°C. Similar trends were also observed in our previous study using a different lytic phages against other serogroups of STEC strains (Liao et al., 2022a). The factors contributing to no bacterial reduction during the period could be due to the equivalent rates of bacterial growth and phage infection. Nevertheless, the bacterial levels continued to drop using higher phage concentrations, MOIs of 100 and 1,000, during the 2-h period. Furthermore, “lysis from without” deriving from high phage concentrations could be the primary cause, resulting in more bacterial reductions than using a low phage dose (MOI = 10) at the 1-h time point (Abedon, 2011). For *E. coli* O157:H7 biofilm, 24- and 48-h biofilm formed in a 24-well plate had weak and moderate biofilm formations, respectively, based on the biofilm validation results (Supplementary Table S3). UDF157lw reduced the OD_590_ of 24- and 48-h *E. coli* O157:H7 biofilm, while the phage without encoding the depolymerase enzyme did not cause any OD_590_ change in 24-h biofilm after 1-h phage treatment. The phenomenon would be likely due to the activity of the depolymerase enzyme to degrade the biofilm EPS, allowing phages to remove the target bacteria in the biofilm (Chang et al., 2022). These findings reveal the potential activity of phage UDF157lw harboring depolymerase enzyme activity to reduce *E. coli* O157:H7 in the early- to mid-stage biofilm *in vitro*. Further studies are necessary to determine the antimicrobial efficacy of mitigating *E. coli* O157:H7 biofilm on different food-contact surfaces.

## 5 Conclusion

The findings of this study reveal that *Escherichia* phage vB_EcoS-UDF157lw (or UDF157lw) is a new member of the *Rogunavirus* phages with a short infection cycle and a large burst size. Without virulent, lysogenic, or antibiotic-resistance genes in the genome, phage UDF157lw could be an alternative antimicrobial agent to combat *E. coli* O157:H7. Most importantly, the phage encodes a prominent depolymerase enzyme with the antimicrobial potential to target *E. coli* O157:H7 in a biofilm formed using a 24-well plate setting. A future study is needed to determine the anti-biofilm efficacy on different surfaces among the applications of individual phages, phage cocktails, and a hurdle intervention.

## Data availability statement

The datasets presented in this study can be found in online repositories. The names of the repository/repositories and accession number(s) can be found in the article/Supplementary material.

## Author contributions

Y-TL: Investigation, Data curation, Formal analysis, Visualization, Writing–original draft. K-JH: Methodology. YZ: Methodology, Writing–review & editing. AS: Methodology, Writing–review & editing. VW: Investigation, Conceptualization, Funding acquisition, Project administration, Resources, Supervision, Writing–review & editing.

## References

[B1] Abedon S. T. (2011). Lysis from without. Bacteriophage 1, 46–49. 10.4161/bact.1.1.13980 21687534 PMC3109453

[B2] Abedon S. T. Herschler T. D. Stopar D. (2001). Bacteriophage latent-period evolution as a response to resource availability. Appl. Environ. Microbiol. 67, 4233–4241. 10.1128/AEM.67.9.4233-4241.2001 11526028 PMC93152

[B3] Bairoch A. Apweiler R. Wu C. H. Barker W. C. Boeckmann B. Ferro S.. (2005). The universal protein resource (UniProt). Nucleic Acids Res. 33, D154–D159. 10.1093/nar/gki070 15608167 PMC540024

[B4] Bortolaia V. Kaas R. S. Ruppe E. Roberts M. C. Schwarz S. Cattoir V.. (2020). ResFinder 4.0 for predictions of phenotypes from genotypes. J. Antimicrob. Chemother. 75, 3491–3500. 10.1093/jac/dkaa345 32780112 PMC7662176

[B5] CDC (2006). Importance of culture confirmation of shiga toxin-producing *Escherichia coli* infection as illustrated by outbreaks of gastroenteritis–New York and North Carolina, 2005. MMWR Morb. Mortal. Wkly. Rep. 55, 1042–1045. Available online at: https://www.cdc.gov/mmwr/preview/mmwrhtml/mm5538a3.htm 17008867

[B6] Chang C. Yu X. Guo W. Guo C. Guo X. Li Q.. (2022). Bacteriophage-mediated control of biofilm: a promising new dawn for the future. Front. Microbiol. 13:825828. 10.3389/fmicb.2022.825828 35495689 PMC9048899

[B7] Chibani-Chennoufi S. Bruttin A. Dillmann M. L. Brüssow H. (2004). Phage-host interaction: an ecological perspective. J. Bacteriophage 186, 3677–3686. 10.1128/JB.186.12.3677-3686.2004 15175280 PMC419959

[B8] Chitlapilly D. S. Bosilevac J. M. Weinroth M. Elowsky C. G. Zhou Y. Anandappa A.. (2020). Impact of mixed biofilm formation with environmental microorganisms on *E. coli* O157, H7. survival against sanitization. NPJ Sci. Food 4:16. 10.1038/s41538-020-00076-x 33083548 PMC7560865

[B9] Clokie M. R. J. Millard A. D. Letarov A. V. Heaphy S. (2011). Phages in nature. Bacteriophage 1, 31–45. 10.4161/bact.1.1.14942 21687533 PMC3109452

[B10] Doolittle M. M. Cooney J. J. Caldwell D. E. (1995). Lytic infection of *Escherichia coli* biofilms by bacteriophage T4. Can. J. Microbiol. 41, 12–18. 10.1139/m95-002 7728652

[B11] Galié S. García-Gutiérrez C. Miguélez E. M. Villar C. J. Lombó F. (2018). Biofilms in the food industry: health aspects and control methods. Front. Microbiol. 9:898. 10.3389/fmicb.2018.00898 29867809 PMC5949339

[B12] Garneau J. R. Depardieu F. Fortier L. C. Bikard D. Monot M. (2017). PhageTerm: a tool for fast and accurate determination of phage termini and packaging mechanism using next-generation sequencing data. Sci. Rep. 7:8292. 10.1038/s41598-017-07910-5 28811656 PMC5557969

[B13] Guo Z. Huang J. Yan G. Lei L. Wang S. Yu L.. (2017). Identification and characterization of Dpo42, a novel depolymerase derived from the *Escherichia coli* phage vB_EcoM_ECOO78. Front. Microbiol. 8:1460. 10.3389/fmicb.2017.01460 28824588 PMC5539073

[B14] Howard-Varona C. Hargreaves K. R. Solonenko N. E. Markillie L. M. White R. A. 3rd Brewer H. M.. (2018). Multiple mechanisms drive phage infection efficiency in nearly identical hosts. ISME J. 12, 1605–1618. 10.1038/s41396-018-0099-8 29568113 PMC5955906

[B15] Hyla K. Dusza I. Skaradzińska A. (2022). Recent advances in the application of bacteriophages against common foodborne pathogens. Antibiotics 11:1536. 10.3390/antibiotics11111536 36358191 PMC9686946

[B16] Jones D. T. Taylor W. R. Thornton J. M. (1992). The rapid generation of mutation data matrices from protein sequences. Comput. Appl. Biosci. 8, 275–282. 10.1093/bioinformatics/8.3.275 1633570

[B17] Kahn L. H. Bergeron G. Bourassa M. W. De Vegt B. Gill J. Gomes F.. (2019). From farm management to bacteriophage therapy: strategies to reduce antibiotic use in animal agriculture. Ann. N. Y. Acad. Sci. 1441, 31-39. 10.1111/nyas.14034 30924542 PMC6850639

[B18] Knecht L. E. Veljkovic M. Fieseler L. (2020). Diversity and function of phage encoded depolymerases. Front. Microbiol. 10:2949. 10.3389/fmicb.2019.02949 31998258 PMC6966330

[B19] Kropinski A. M. Lingohr E. J. Moyles D. M. Ojha S. Mazzocco A. She Y.-M.. (2012). Endemic bacteriophages: a cautionary tale for evaluation of bacteriophage therapy and other interventions for infection control in animals. Virol. J. 9:207. 10.1186/1743-422X-9-207 22985539 PMC3496638

[B20] Kuruwita D. P. Jiang X. Darby D. Sharp J. L. Fraser A. M. (2022). Persistence of *Escherichia coli* O157, H7. and *Listeria monocytogenes* on the exterior of three common food packaging materials. Food Control. 112:107153. 10.1016/j.foodcont.2020.107153

[B21] Lacombe A. Quintela I. A. Liao Y.-T. Wu V. C. H. (2022). Shiga toxin-producing *Escherichia coli* outbreaks in California's leafy greens production continuum. Front. Food Sci. Technol. 2:1068690. 10.3389/frfst.2022.1068690

[B22] León M. Bastías R. (2015). Virulence reduction in bacteriophage resistant bacteria. Front. Microbiol. 6:343. 10.3389/fmicb.2015.00343 25954266 PMC4407575

[B23] Liao Y.-T. Lavenburg V. M. Lennon M. Salvador A. Hsu A. L. Wu V. C. H.. (2020). The effects of environmental factors on the prevalence and diversity of bacteriophages lytic against the top six non-O157 Shiga toxin-producing *Escherichia coli* on an organic farm. J. Food Saf. 43:e12865. 10.1111/jfs.12865

[B24] Liao Y.-T. Salvador A. Harden L. A. Liu F. Lavenburg V. M. Li R. W.. (2019). Characterization of a lytic bacteriophage as an antimicrobial agent for biocontrol of shiga toxin-producing *Escherichia coli* O145 strains. Antibiotics 8:74. 10.3390/antibiotics8020074 31195679 PMC6627115

[B25] Liao Y.-T. Zhang Y. Salvador A. Harden L. A. Wu V. C. H. (2022a). Characterization of a T4-like bacteriophage vB_EcoM-Sa45lw as a potential biocontrol agent for shiga toxin-producing *Escherichia coli* O45 contaminated on mung bean seeds. Microbiol. Spectr. 10:e02220-21. 10.1128/spectrum.02220-21 35107386 PMC8809338

[B26] Liao Y.-T. Zhang Y. Salvador A. Ho K. J. Cooley M. B. Wu V. C. H.. (2022b). Characterization of polyvalent *Escherichia* phage Sa157lw for the biocontrol potential of *Salmonella* Typhimurium and *Escherichia coli* O157:H7 on contaminated mung bean seeds. Front. Microbiol. 13:1053583. 10.3389/fmicb.2022.1053583 36439834 PMC9686305

[B27] Lowe T. M. Chan P. P. (2016). tRNAscan-SE On-line: integrating search and context for analysis of transfer RNA genes. Nucleic Acids Res. 44, W54–W57. 10.1093/nar/gkw413 27174935 PMC4987944

[B28] Łusiak-Szelachowska M. Weber-Dabrowska B. Górski A. (2020). Bacteriophages and lysins in biofilm control. Virol. Sin. 35, 125–133. 10.1007/s12250-019-00192-3 32125643 PMC7198674

[B29] Mahadevan P. King J. F. Seto D. (2009). CGUG: *in silico* proteome and genome parsing tool for the determination of “core” and unique genes in the analysis of genomes up to ca. 1.9 Mb. BMC Res. Methods 2:168. 10.1186/1756-0500-2-168 19706165 PMC2738686

[B30] Malberg Tetzschner A. M. Johnson J. R. Johnston B. D. Lund O. Scheutz F. (2020). *In Silico* genotyping of *Escherichia coli* isolates for extraintestinal virulence genes by use of whole-genome sequencing data. J. Clin. Microbiol. 58, e0126920. 10.1128/JCM.01269-20 32669379 PMC7512150

[B31] Marouani-Gadri N. Firmesse O. Chassaing D. Sandris-Nielsen D. Arneborg N. Carpentier B.. (2010). Potential of *Escherichia coli* O157:H7 to persist and form viable but non-culturable cells on a food-contact surface subjected to cycles of soiling and chemical treatment. Int. J. Food Microbiol. 144, 96–103. 10.1016/j.ijfoodmicro.2010.09.002 20888655

[B32] Meier-Kolthoff J. P. Göker M. (2017). VICTOR: genome-based phylogeny and classification of prokaryotic viruses. Bioinformatics 33, 3396–3404. 10.1093/bioinformatics/btx440 29036289 PMC5860169

[B33] Merabishvili M. Vervaet C. Pirnay J.-P. De Vos D. Verbeken G. Mast J.. (2013). Stability of *Staphylococcus aureus* phage ISP after freeze-drying (lyophilization). PLoS ONE 8:e68797. 10.1371/journal.pone.0068797 23844241 PMC3699554

[B34] Nilsson A. S. (2014). Phage therapy–constraints and possibilities. Ups. J. Med. Sci. 119, 192–198. 10.3109/03009734.2014.902878 24678769 PMC4034558

[B35] Niu Y. D. McAllister T. A. Nash J. H. Kropinski A. M. Stanford K. (2014). Four *Escherichia coli* O157:H7 phages: a new bacteriophage genus and taxonomic classification of T1-like phages. PLoS ONE 9, e100426. 10.1371/journal.pone.0100426 24963920 PMC4070988

[B36] Nobrega F. L. Vlot M. de Jonge P. A. Dreesens L. L. Beaumont H. J. E. Lavigne R.. (2018). Targeting mechanisms of tailed bacteriophages. Nat. Rev. Microbiol. 16, 760–773. 10.1038/s41579-018-0070-8 30104690

[B37] Oliveira L. Tavares P. Alonso J. C. (2013). Headful DNA packaging: bacteriophage SPP1 as a model system. Virus Res. 173, 247–259. 10.1016/j.virusres.2013.01.021 23419885

[B38] O'Sullivan L. Bolton D. McAuliffe O. Coffey A. (2019). Bacteriophages in food applications: from foe to friend. Annu. Rev. Food Sci. Technol. 10, 151–172. 10.1146/annurev-food-032818-121747 30633564

[B39] Park D. W. Park J. H. (2021). Characterization and food application of the novel lytic phage BECP10: specifically recognizes the O-polysaccharide of *Escherichia coli* O157, H7. Viruses 13:1469. 10.3390/v13081469 34452334 PMC8402813

[B40] Partanen P. Hultman J. Paulin L. Auvinen P. Romantschuk M. (2010). Bacterial diversity at different stages of the composting process. BMC Microbiol. 10:94. 10.1186/1471-2180-10-94 20350306 PMC2907838

[B41] Pinto G. Almeida C. Azeredo J. (2020). Bacteriophages to control Shiga toxin-producing *E. coli*- safety and regulatory challenges. Crit. Rev. Biotechnol. 40, 1081–1097. 10.1080/07388551.2020.1805719 32811194

[B42] Pinto G. Minnich S. A. Hovde C. J. Oliveira H. Smidt H. Almeida C.. (2021). The interactions of bacteriophage Ace and Shiga toxin-producing *Escherichia coli* during biocontrol. FEMS Microbiol. Ecol. 97:fiab105. 10.1093/femsec/fiab105 34329454 PMC8492476

[B43] Połaska M. Sokołowska B. (2019). Bacteriophages - a new hope or a huge problem in the food industry. AIMS Microbiol. 5, 324–346. 10.3934/microbiol.2019.4.324 31915746 PMC6946638

[B44] Rahal E. A. Kazzi N. Nassar F. J. Matar G. M. (2012). *Escherichia coli* O157, H7.—clinical aspects and novel treatment approaches. Front. Cell. Infect. Microbiol. 2:138. 10.3389/fcimb.2012.00138 23162800 PMC3498739

[B45] Richter M. Rosselló-Móra R. Oliver Glöckner F. Peplies J. (2016). JSpeciesWS: a web server for prokaryotic species circumscription based on pairwise genome comparison. Bioinformatics 32, 929–931. 10.1093/bioinformatics/btv681 26576653 PMC5939971

[B46] Riley L. W. Remis R. S. Helgerson S. D. McGee H. B. Wells J. G. Davis B. R.. (1983). Hemorrhagic colitis associated with a rare *Escherichia coli* serotype. N. Engl. J. Med. 308, 681–685. 10.1056/NEJM198303243081203 6338386

[B47] Seemann T. (2014). Prokka: rapid prokaryotic genome annotation. Bioinformatics 30, 2068–2069. 10.1093/bioinformatics/btu153 24642063

[B48] Son H. M. Duc H. M. Masuda Y. Honjoh K. I. Miyamoto T. (2018). Application of bacteriophages in simultaneously controlling *Escherichia coli* O157:H7 and extended-spectrum beta-lactamase producing *Escherichia coli*. Appl. Microbiol. Biotechnol. 102,10259–10271. 10.1007/s00253-018-9399-1 30267128

[B49] Sørensen A. N. Woudstra C. Sørensen M. C. H. Brøndsted L. (2021). Subtypes of tail spike proteins predicts the host range of *Ackermannviridae* phages. Comput. Struct. Biotechnol. J. 19, 4854–4867. 10.1016/j.csbj.2021.08.030 34527194 PMC8432352

[B50] Stepanovic S. Vukovic D. Dakic I. Savic B. Svabic-Vlahovic M. (2000). A modified microtiter-plate test for quantification of staphylococcal biofilm formation. J. Microbiol. Methods. 40, 175–179. 10.1016/S0167-7012(00)00122-6 10699673

[B51] Tack D. M. Kisselburgh H. M. Richardson L. C. Geissler A. Griffin P. M. Payne D. C.. (2021). Shiga toxin-producing *Escherichia coli* outbreaks in the United States, 2010-2017. Microorganisms 9, 1529. 10.3390/microorganisms9071529 34361964 PMC8307841

[B52] Turner D. Kropinski A. M. Adriaenssens E. M. (2021). A roadmap for genome-based phage taxonomy. Viruses 13:506. 10.3390/v13030506 33803862 PMC8003253

[B53] Uhlich G. A. Paoli G. C. Zhang X. Andreozzi E. (2019). Whole-genome sequence of *Escherichia coli* serotype O157:H7 strain ATCC 43888. Microbiol. Resour. Announc. 8:e00906-19. 10.1128/MRA.00906-19 31624165 PMC6797530

[B54] Vikram A. Tokman J. I. Woolston J. Sulakvelidze A. (2020). Phage biocontrol improves food safety by significantly reducing the level and prevalence of *Escherichia coli* O157:H7 in various foods. J. Food Prot. 83, 668–676. 10.4315/0362-028X.JFP-19-433 32221572

[B55] Wu V. C. (2008). A review of microbial injury and recovery methods in food. Food Microbiol. 25, 735–744. 10.1016/j.fm.2008.04.011 18620965

[B56] Zhang Y. Liao Y.-T. Salvador A. Lavenburg V. M. Wu V. C. H. (2021). Characterization of two new shiga toxin-producing *Escherichia coli* O103-infecting phages isolated from an organic farm. Microorganisms 9:1527. 10.3390/microorganisms9071527 34361962 PMC8303462

