# Supplementary material for: A new Rogue-like Escherichia phage UDF157lw to control Escherichia coli O157:H7
Source: Front Microbiol. 2024 Jan 22;14:1302032. doi: 10.3389/fmicb.2023.1302032 (PMC10838988; doi:10.3389/fmicb.2023.1302032)
Supplement: Supplementary file 2 [file Data_Sheet_1.docx]

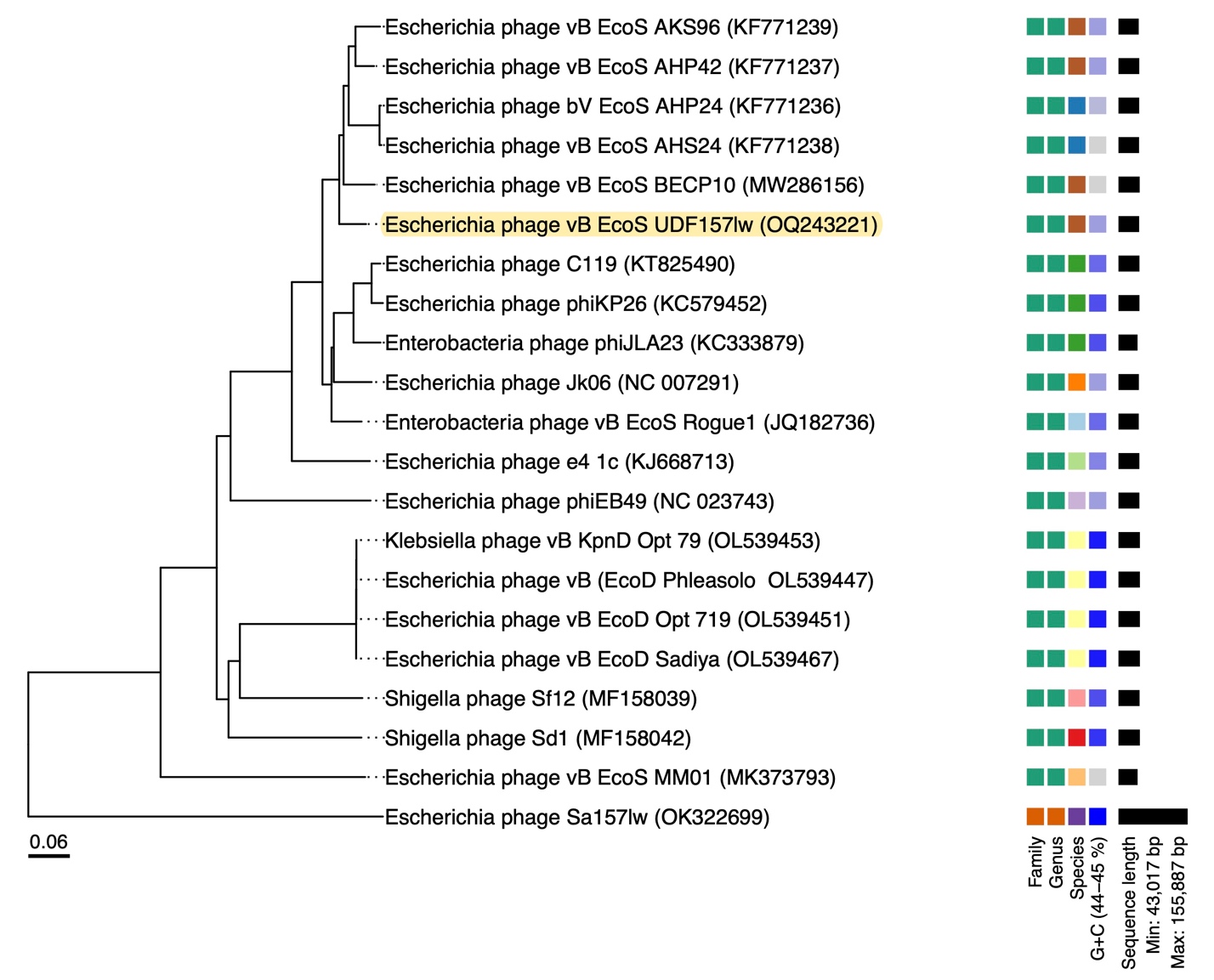


**Figure S1**. Whole genome sequence phylogenetic tree constructed by VICTOR based on the amino acid (formula d6) of UDF157lw, other *Caudoviricetes* phage Sa157lw, and the close-related reference phages belonging to the *Rogunaviru* genus under the *Drexlerviridae* family. The family, genus, and species are classified into different clusters with colors based on VICTOR analysis. Genomic GC content and sequence length are represented in the hue of color and horizontal black lines, respectively, on the right side of the tree.


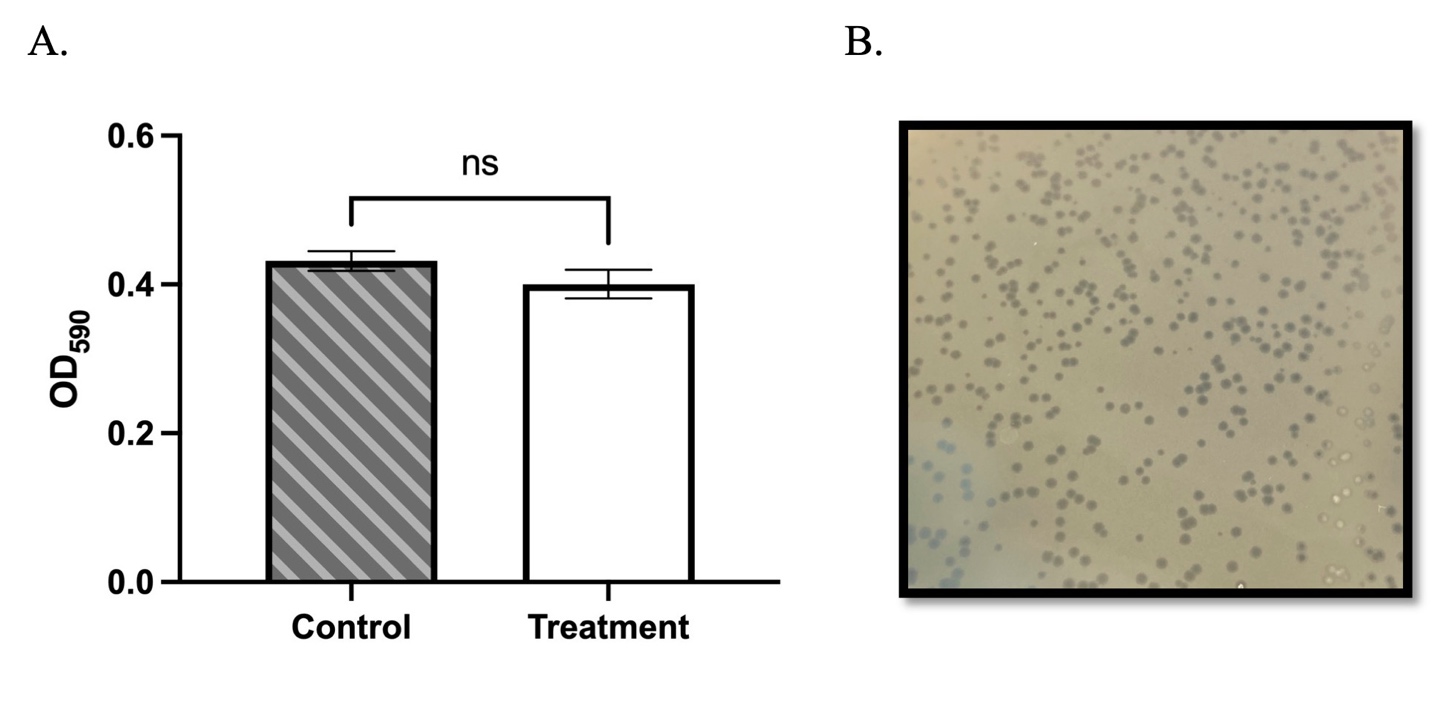


**Figure S2**. Antimicrobial activity of phage A against *E. coli* O157:H7 (RM9995) biofilm at 37 °C for 1 h based on the bacterial optical density at OD_590_ (A), and the plaque morphology of phage A on a plaque-assay plate with top soft agar (B). ns indicates no significant difference between the control and treatment groups. The error bars show the standard error of the mean (SEM).
